# Critical Tracheal Compression by a Double Aortic Arch in a Young Infant: A Case Report

**DOI:** 10.7759/cureus.116703

**Published:** 2026-09-22

**Authors:** Kabelo Molatedi, Mikail Amod, Jeandre Kotze, Ambrosius Swartbooi

**Affiliations:** 1 Department of Radiology, Robert Mangaliso Sobukwe Hospital, Kimberley, ZAF; 2 Department of Orthopaedic Surgery, Frere Tertiary Hospital, East London, ZAF; 3 Department of Orthopaedic Surgery, Groote Schuur Hospital, Cape Town, ZAF

**Keywords:** airway obstruction, computed tomography angiography, double aortic arch, infant, stridor, tracheal compression, vascular ring

## Abstract

Double aortic arch is a congenital vascular anomaly in which persistence of both fourth aortic arches creates a complete vascular ring around the trachea and esophagus. Severe airway compromise in early infancy may be life-threatening and can mimic intrinsic congenital airway disease. We report a six-week-old female infant with progressive noisy breathing, stridor, and respiratory distress, particularly during crying, who experienced three near-arrest episodes. Computed tomography angiography demonstrated a complete vascular ring formed by a double aortic arch with an atretic left arch and severe focal tracheal compression. The narrowest tracheal diameter measured 1.7 mm across a 5.1-mm segment at the T1/T2 level, with associated posterior esophageal compression. The right common carotid and right subclavian arteries arose from the right arch, whereas the left vertebral, left common carotid, and left subclavian arteries arose from the left arch. The infant underwent thoracotomy and release of the vascular ring. Respiratory distress and stridor improved markedly after surgery, and she was discharged breathing room air without stridor at rest and feeding well. The imaging findings and postoperative response supported extrinsic vascular compression rather than documented intrinsic congenital tracheal stenosis or a tracheal web. Recognition of the characteristic vascular anatomy is essential because double aortic arch, pulmonary artery sling, and intrinsic congenital tracheal stenosis have different anatomic substrates and surgical implications.

## Introduction

Vascular rings are congenital abnormalities of the aortic arch and its derivatives in which vascular or ligamentous structures encircle and/or externally compress the trachea, esophagus, or both. They are uncommon but clinically important because respiratory and feeding manifestations can be nonspecific. Symptomatic infants may present with stridor, noisy breathing, wheezing, recurrent respiratory infections, apnea, cyanotic episodes, respiratory distress, dysphagia, or feeding difficulty. In young infants, even marked reductions in airway caliber may produce substantial respiratory compromise because of the small baseline airway diameter [1-4].

Double aortic arch is a true complete vascular ring produced by persistence of both right and left fourth aortic arch components and associated dorsal aortic segments. The two arches course on opposite sides of the trachea and esophagus and join posteriorly to form the descending aorta. A right-dominant configuration is commonly reported, although codominant and left-dominant configurations can occur; an atretic segment may also be present [1-3]. Double aortic arch is an important cause of symptomatic vascular-ring disease in infancy and may present early when airway compression is severe [3,4].

The distinction between extrinsic vascular compression and intrinsic congenital tracheal stenosis is particularly important when the airway lumen is critically narrowed, because the two mechanisms have different anatomical causes and management strategies. This distinction has direct clinical implications because extrinsic vascular compression may be treated by release of the vascular ring, whereas intrinsic congenital tracheal stenosis may require airway-directed intervention. Congenital tracheal stenosis is most commonly related to complete cartilaginous tracheal rings and represents a primary abnormality of the tracheal wall. By contrast, vascular rings cause secondary airway narrowing through external compression. When the luminal reduction is extreme, the finding of severe tracheal narrowing on imaging does not by itself establish whether the underlying mechanism is intrinsic tracheal disease or extrinsic vascular compression [5,6].

Pulmonary artery sling is another important vascular cause of severe infantile airway obstruction. In pulmonary artery sling, the left pulmonary artery arises anomalously from the right pulmonary artery and passes between the trachea and esophagus. This anomaly has a recognized association with congenital tracheal stenosis and complete tracheal rings, making differentiation from double aortic arch clinically important [7,8].

Computed tomography angiography can provide rapid, high-resolution assessment of aortic arch configuration, branch-vessel origins, the relationship of vascular structures to the trachea and esophagus, and the location and severity of airway compression. These features are particularly useful for surgical planning [1,2]. The present case illustrates the diagnostic distinction between a suspected intrinsic distal tracheal lesion and critical extrinsic airway compression caused by a double aortic arch, emphasizing the importance of early recognition and multidisciplinary evaluation in an infant with unexplained stridor.

## Case presentation

Clinical presentation

A term female infant was born before arrival on June 17, 2026, at a birth weight of 3,540 g. No major birth complications were documented. She was HIV-exposed; the maternal viral load on the day of delivery was 517 copies/mL, the birth HIV polymerase chain reaction (PCR) was negative, and she received high-risk prophylaxis with zidovudine and nevirapine. Immunizations were reported to be up to date.

Noisy breathing was reported from birth and progressively worsened during the first weeks of life. The infant developed prominent inspiratory and later biphasic stridor. Her mother reported that she could feed only for short periods before pausing to breathe. There was no reported fever, recent respiratory infection, or trauma. At approximately five weeks of age, the infant was assessed by otorhinolaryngology. Flexible nasopharyngolaryngoscopy showed patent bilateral nostrils and normal structures above the vocal cords; laryngomalacia was excluded. The combination of persistent biphasic stridor, head bobbing, and accessory muscle use raised concern for a lower airway process.

At Robert Mangaliso Sobukwe Hospital, a tertiary hospital, the infant remained initially stable enough to feed but continued to have noisy breathing. On July 22, 2026 (day 35 of life), computed tomography (CT) of the neck and chest was interpreted as showing features highly suggestive of a tracheal web at the level of the carina. The examination also demonstrated a right-sided thoracic aorta, and dedicated CT angiography was recommended to clarify the arch anatomy.

On July 24, 2026 (day 37 of life), CT angiography demonstrated abnormal bilateral aortic arch anatomy. The right-sided arch gave rise to the right common carotid and right subclavian arteries, while a left-sided arch gave rise to the left common carotid and left subclavian arteries and contained an atretic segment. The vascular configuration produced mass effect on the distal trachea with significant airway stenosis. This changed the working diagnosis from a possible intrinsic tracheal lesion to a vascular cause of airway compression.

The infant remained under pediatric care while arrangements were made for specialist management. Concern regarding poor weight gain was documented, with an estimated gain of approximately 40 g per week. On July 27, 2026 (day 40 of life), she was transferred from the Paediatric Department of Robert Mangaliso Sobukwe Hospital to the Paediatric Cardiology service at Universitas Academic Hospital for further assessment and management.

Clinical deterioration after transfer

Following transfer, the infant developed progressively worsening respiratory obstruction, particularly with crying. She developed marked supraclavicular and subcostal recession and tracheal tug and required high-flow oxygen. Three near-arrest episodes occurred in association with crying and worsening airway obstruction, prompting pediatric intensive care management and urgent cardiothoracic surgical assessment. There was no documented cardiac murmur, and the available cardiac imaging did not demonstrate a major intracardiac shunt or other major structural cardiac defect.

Diagnostic imaging

CT heart angiography performed on July 29, 2026 (day 42 of life) demonstrated a double aortic arch with an atretic left arch, forming a complete vascular ring around the trachea and esophagus. The right common carotid and right subclavian arteries arose from the right arch, whereas the left vertebral, left common carotid, and left subclavian arteries arose from the left arch. The vascular ring compressed the trachea anteriorly and the esophagus posteriorly (Figure 1).

**Figure 1:**
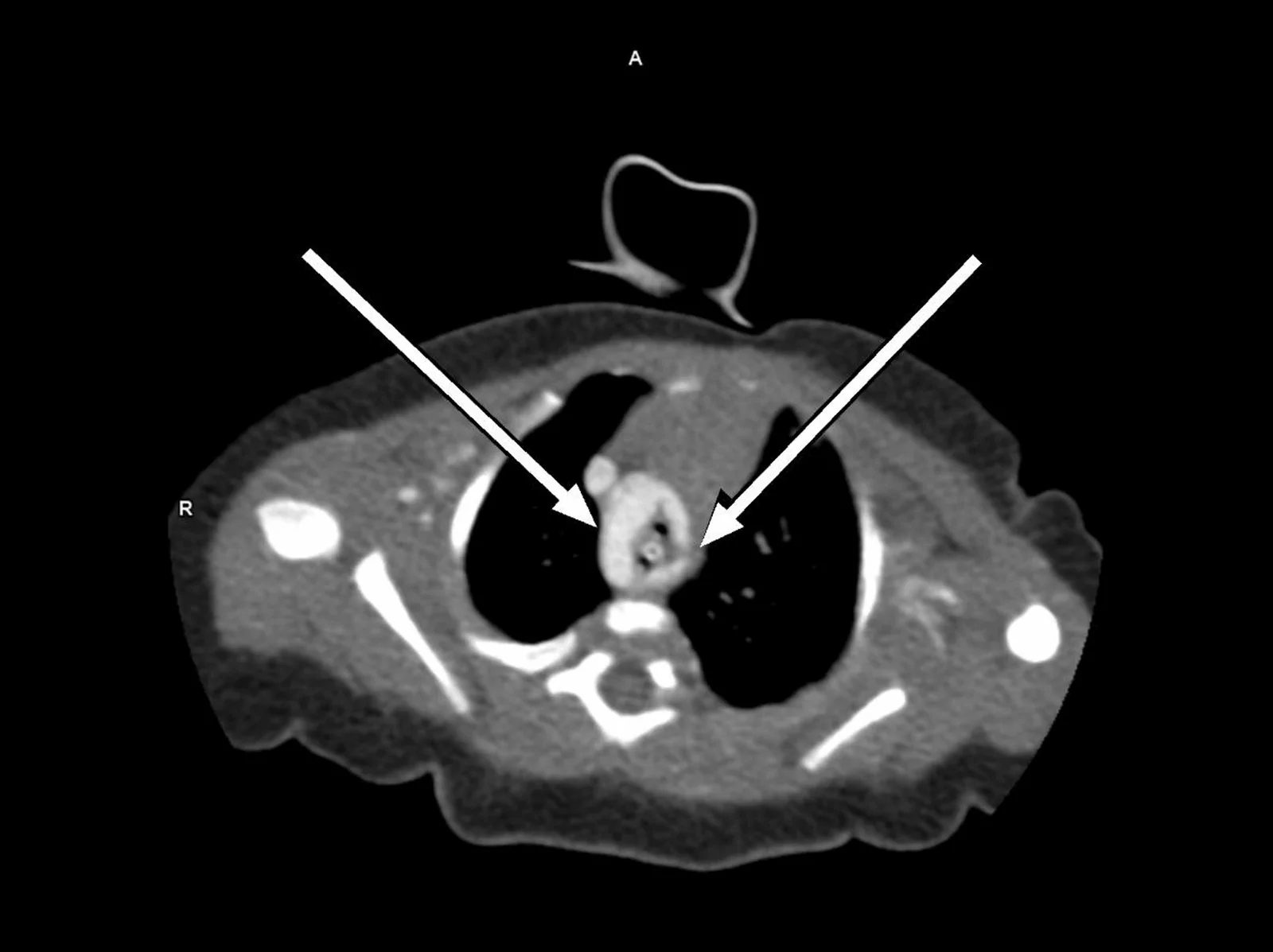
Axial computed tomography angiography showing the complete vascular ring The white arrows indicate the right and left aortic arches coursing on either side of the centrally located trachea and esophagus, producing circumferential vascular encirclement.

The critical airway narrowing was located at approximately the T1/T2 vertebral level. The minimum reported tracheal diameter was 1.7 mm across a narrowed segment measuring approximately 5.1 mm. The lungs were reported as normal. Cardiac findings included a single superior vena cava draining to the right atrium, normal inferior vena caval drainage, no atrial or ventricular septal defect, normal right ventricular morphology, normal pulmonary venous drainage, a dilated left atrium, and a normal-sized left ventricle. No pericardial effusion was reported (Figure 2).

**Figure 2:**
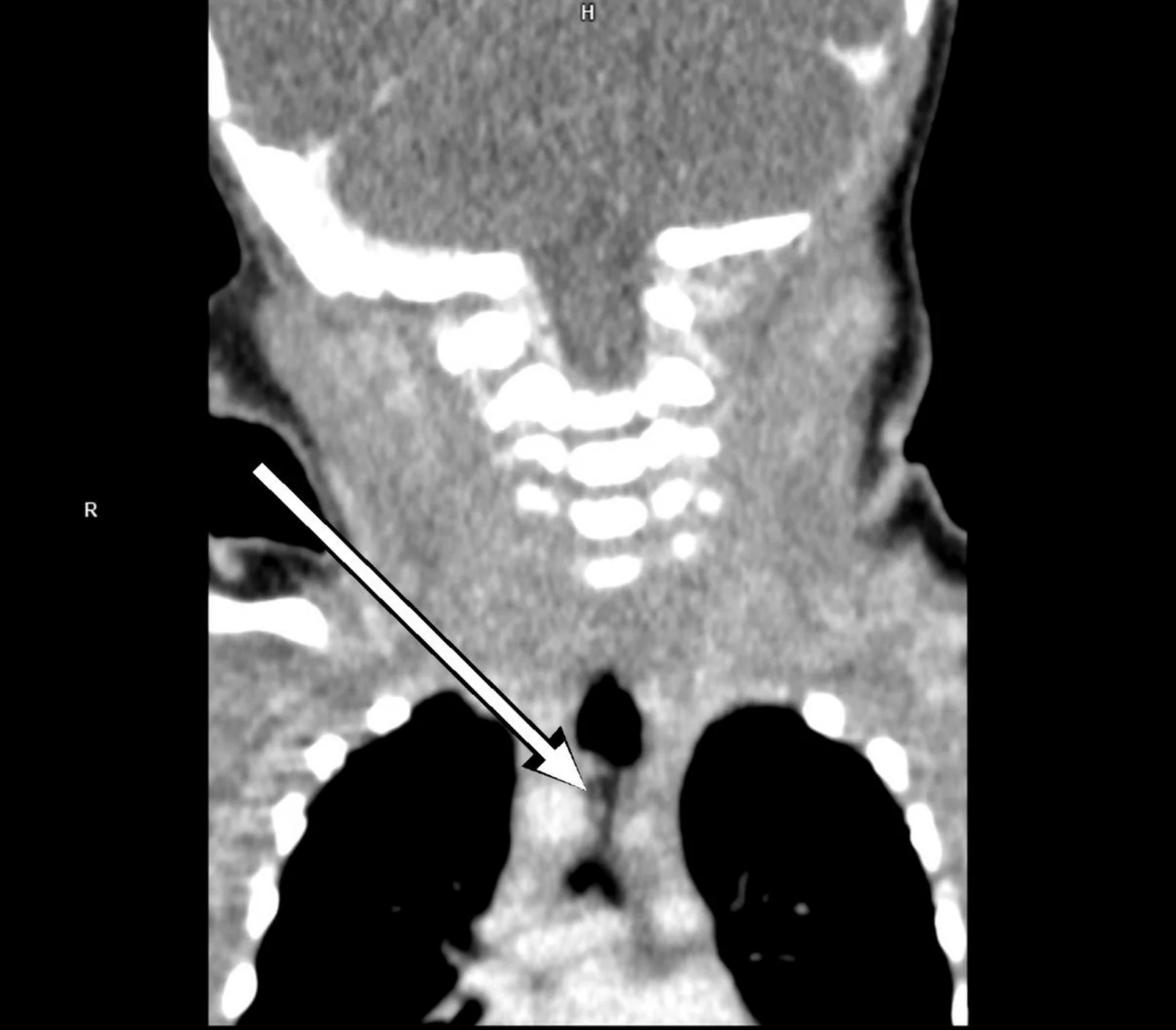
Coronal computed tomography angiography showing critical focal tracheal narrowing The white arrow identifies the critically narrowed tracheal segment at approximately the T1/T2 level. The reported minimum tracheal diameter is 1.7 mm across a 5.1-mm segment.

The sequence of imaging was clinically important. The initial CT raised concern for a focal intrinsic distal tracheal lesion, whereas subsequent dedicated angiographic imaging demonstrated the complete vascular ring responsible for the airway compression. The available imaging did not demonstrate a tracheal web or complete tracheal rings. No bronchoscopy report was available; therefore, subtle intrinsic tracheal abnormalities cannot be excluded with absolute certainty (Figure 3).

**Figure 3:**
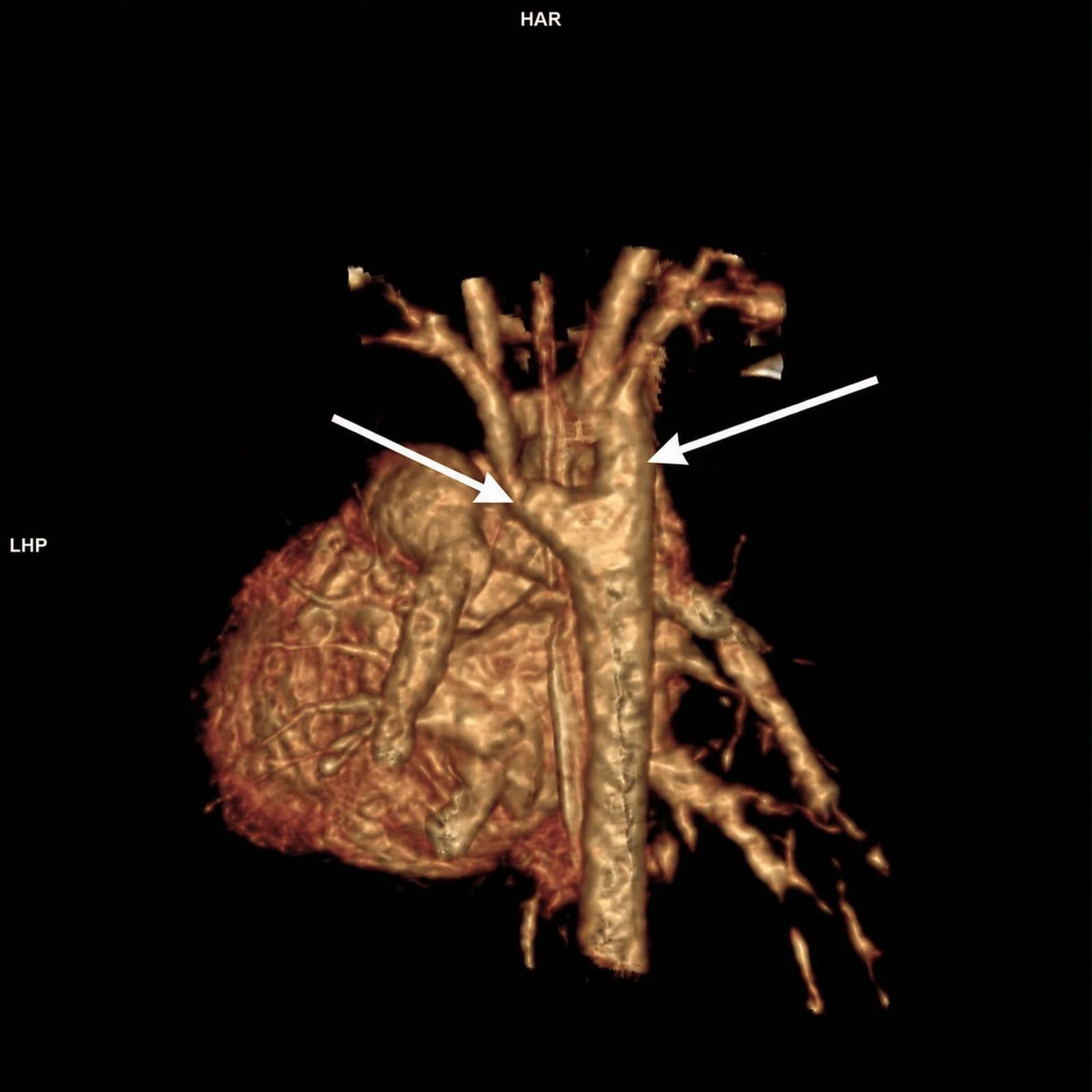
Three-dimensional volume-rendered computed tomography angiography demonstrating the double aortic arch The white arrows indicate the two limbs of the double aortic arch. The volume-rendered reconstruction demonstrates the associated branch-vessel anatomy and complete vascular ring configuration. Three-dimensional reconstruction exported from the software Syngo.via VB80 (Siemens Healthineers AG, Erlangen, Germany) at Robert Mangaliso Sobukwe Hospital by Dr. Kabelo Molatedi.

Laboratory investigations

The available laboratory results did not demonstrate a significant inflammatory or infectious process. All values from the supplied Robert Mangaliso Sobukwe Hospital/Kimberley Laboratory reports dated July 22 and 27, 2026 are summarized in Table 1, with the laboratory-specific reference intervals shown where provided. The potassium result on July 22 was rejected because of haemolysis and therefore has not been interpreted.

**Table 1:** Laboratory findings during hospitalization. Reference intervals in this table are printed on the National Health Laboratory Service reports. The differential leukocyte reference intervals shown are the laboratory's absolute-count intervals; percentage reference intervals were not provided on the report.

Day	Investigation	Result	Laboratory reference interval
D35	Sodium	132 mmol/L (low)	136-145 mmol/L
D35	Potassium	Rejected; result invalid due to haemolysis	Not reported
D35	Urea	1.4 mmol/L	1.4-5.0 mmol/L
D35	Creatinine	21 µmol/L	10-56 µmol/L
D35	C-reactive protein	<1 mg/L	<10 mg/L
D35	White cell count	12.23 ×10⁹/L	5.00-19.50×10⁹/L
D35	Red cell count	4.18 ×10¹²/L	3.90-5.30×10¹²/L
D35	Haemoglobin	15.1 g/dL (high)	10.0-15.0 g/dL
D35	Haematocrit	0.419 L/L	0.290-0.440 L/L
D35	Mean corpuscular volume	100.2 fL	86.0-124.0 fL
D35	Mean corpuscular haemoglobin	36.1 pg (high)	29.0-36.0 pg
D35	Mean corpuscular haemoglobin concentration	36.0 g/dL	28.0-36.0 g/dL
D35	Red cell distribution width	13.9 %	Not reported
D35	Platelet count	361×10⁹/L (high)	140-350×10⁹/L
D35	Mean platelet volume	9.9 fL	7.0-11.4 fL
D35	Neutrophils	6.50 %; 0.79×10⁹/L	Absolute: 2.00-8.00×10⁹/L
D35	Lymphocytes	74.80 %; 9.15×10⁹/L	Absolute: 2.00-17.00×10⁹/L
D35	Monocytes	6.90 %; 0.84×10⁹/L	Absolute: 0.50-1.80×10⁹/L
D35	Eosinophils	11.00 %; 1.35×10⁹/L	Absolute: 0.20-0.80×10⁹/L
D35	Basophils	0.40 %; 0.05×10⁹/L	Absolute: 0.00-0.20×10⁹/L
D35	Immature cells	0.00 %; 0.0×10⁹/L	Not reported
D40	Sodium	138 mmol/L	136-145 mmol/L
D40	Potassium	5.6 mmol/L (high)	4.1-5.3 mmol/L
D40	Urea	2.6 mmol/L	1.4-5.0 mmol/L
D40	Creatinine	22 µmol/L	10-56 µmol/L
D40	Calcium	2.54 mmol/L	2.22-2.62 mmol/L
D40	Magnesium	0.89 mmol/L	0.78-0.99 mmol/L
D40	Inorganic phosphate	2.14 mmol/L (high)	1.20-2.10 mmol/L
D40	C-reactive protein	<1 mg/L	<10 mg/L

Surgical treatment and postoperative course

Because of severe symptomatic airway obstruction and recurrent near-arrest episodes, urgent cardiothoracic surgical management was undertaken. On July 30, 2026 (day 43 of life), the infant underwent thoracotomy and release of the vascular ring. The available operative documentation did not specify the surgical side or the individual vascular structure divided; these details were therefore not inferred in this report.

An intercostal drain was placed and initially drained serous fluid. Following vascular-ring release, stridor and work of breathing improved markedly. High-flow oxygen was initially continued after surgery and was subsequently weaned to nasal oxygen. The intercostal drain was removed on August 2, 2026 (postoperative day four), and the infant was breathing room air by August 3, 2026. She was discharged on August 4, 2026 (postoperative day five), breathing room air, without stridor at rest, and breastfeeding well. Follow-up with pediatric, pulmonology, and otorhinolaryngology services was planned.

Clinical timeline

The clinical timeline of the infant's condition is presented in Table 2.

**Table 2:** Clinical timeline from birth to discharge. ENT: Ear, nose, throat; PCR: polymerase chain reaction

Time	Clinical course and key investigations
D0 - June 17, 2026	Term female infant born before arrival; birth weight 3,540 g. HIV-exposed; birth HIV PCR negative.
D0-D35	Noisy breathing/stridor present from birth and progressively worsening; feeding pauses required to breathe.
D35 - July 22	ENT assessment demonstrated biphasic stridor; flexible examination showed normal structures above the vocal cords and laryngomalacia was excluded. Initial CT suggested a distal tracheal abnormality and right-sided thoracic aorta.
D37 - July 24	CT angiography demonstrated bilateral arch anatomy with an atretic left arch and significant distal tracheal compression.
D40 - July 27	Transferred from Robert Mangaliso Sobukwe Hospital Paediatrics to Universitas Academic Hospital Paediatric Cardiology.
D40-D42 - July 27-29	Progressive respiratory distress with supraclavicular/subcostal recession and three near-arrest episodes triggered by crying; high-flow oxygen required.
D42 - July 29	CT heart angiography confirmed a complete vascular ring due to a double aortic arch with an atretic left arch; minimum tracheal diameter 1.7 mm over 5.1 mm at T1/T2.
D43 - July 30	Thoracotomy and release of the vascular ring.
D46 - August 2	Intercostal drain removed.
D47 - August 3	Weaned to room air.
D48 - August 4	Discharged breathing room air, without stridor at rest and breastfeeding well.

## Discussion

Double aortic arch as a cause of critical airway obstruction

Double aortic arch is a true complete vascular ring in which persistent right and left arch components pass on either side of the trachea and esophagus before joining to form the descending aorta [1-4]. It is an important cause of symptomatic vascular-ring disease in infancy, and neonatal or early-infant presentations with respiratory compromise have been described [3,4,9]. The present infant developed stridor from birth and subsequently progressed to severe airway obstruction, including near-arrest episodes during crying.

The clinical pattern was strongly suggestive of severe vascular-ring-related airway obstruction. Symptoms of vascular rings include stridor, wheezing, recurrent respiratory infection, apnea, cyanosis, respiratory distress, and feeding difficulty, with severity influenced by the vascular configuration and degree of airway compression [1-4]. In this case, the upper-airway examination was unrevealing and laryngomalacia was excluded, increasing suspicion for a lower airway or mediastinal cause.

The most striking imaging feature was the degree of tracheal narrowing. The reported minimum tracheal diameter on CT angiography was 1.7 mm across a 5.1-mm segment at T1/T2. The available radiology documentation did not specify the measurement technique. Such a critically reduced airway caliber provides a plausible anatomical explanation for the dramatic clinical deterioration with crying, when respiratory demand increases. Previous physiologic work in infants with vascular rings has demonstrated severe airway dysfunction before repair and improvement after vascular-ring surgery, although abnormalities may persist during the early postoperative period [10]. Prolonged vascular compression has been associated with persistent airway abnormalities, including tracheomalacia, which may contribute to residual respiratory symptoms after repair [10,11].

Extrinsic vascular compression versus intrinsic congenital tracheal stenosis

The distinction between extrinsic vascular compression and intrinsic congenital tracheal stenosis was central to this case. Congenital tracheal stenosis is most commonly caused by complete cartilaginous tracheal rings and may produce short- or long-segment narrowing. The severity of symptoms is influenced by the degree and length of narrowing and by associated airway or cardiopulmonary abnormalities [5,6]. Bronchoscopy is particularly useful when direct assessment of the internal tracheal morphology is required.

The initial CT examination in this case was interpreted as highly suggestive of a tracheal web at the level of the carina. However, subsequent dedicated CT angiography demonstrated a complete vascular ring at the same level as the critical airway narrowing, with the right and left arches encircling the trachea and esophagus. The imaging reports available to the authors did not demonstrate a tracheal web, complete tracheal rings, or another primary intrinsic tracheal abnormality.

The term 'critical tracheal stenosis' should therefore be interpreted carefully in this case. It accurately describes the severity of the reduced tracheal lumen but does not, by itself, establish a primary intrinsic tracheal stenosis. The strongest evidence for the mechanism is the combination of direct vascular encirclement at the level of narrowing, absence of a documented intrinsic lesion on available imaging, and marked clinical improvement following vascular-ring release. Because bronchoscopy was not available, however, a subtle intrinsic airway abnormality cannot be excluded with absolute certainty.

This distinction has direct management implications. Symptomatic double aortic arch is treated by surgical division and release of the vascular ring, whereas primary congenital tracheal stenosis caused by complete rings may require airway reconstruction, including slide tracheoplasty or other reconstructive approaches in selected patients [5,6].

Differentiating double aortic arch from pulmonary artery sling

Pulmonary artery sling is an important alternative vascular cause of severe infantile airway obstruction. In this anomaly, the left pulmonary artery arises from the right pulmonary artery and passes posterior to the trachea and anterior to the esophagus before reaching the left hilum. Pulmonary artery sling is strongly associated with congenital tracheal stenosis and complete tracheal rings [7,8].

In the present case, CT angiography directly demonstrated two aortic arches forming a complete vascular ring and identified the branch-vessel origins from each arch. No anomalous left pulmonary artery arising from the right pulmonary artery was reported. The imaging anatomy therefore supports double aortic arch rather than pulmonary artery sling. This distinction is important because the two anomalies have different vascular anatomy, patterns of airway involvement, and operative strategies.

Role of computed tomography angiography and early recognition

CT angiography was decisive in this case because it simultaneously characterized the aortic arch configuration, branch-vessel origins, relationship of the vascular structures to the trachea and esophagus, and the location and severity of airway compression. Contemporary imaging reviews describe CT as a preferred modality for vascular rings because rapid acquisition permits high spatial resolution with multiplanar and three-dimensional reconstructions and simultaneous assessment of the airway [1,2].

The diagnostic sequence illustrates an important practical point. When CT demonstrates focal distal tracheal narrowing together with abnormal mediastinal vascular anatomy, dedicated angiographic imaging should be considered before attributing the stenosis to a primary intrinsic airway lesion. In this infant, the subsequent CTA changed the diagnostic interpretation and provided anatomy relevant to surgical planning.

Early recognition is particularly important when symptoms are progressive or are provoked by crying and feeding. Published neonatal cases demonstrate that double aortic arch can present very early with severe respiratory compromise [4,9]. Airway management in affected infants may also be challenging because of the critical distal airway compression [12]. In the present case, the combination of persistent stridor, a normal upper-airway examination, progressive work of breathing, and critical distal tracheal narrowing warranted rapid multidisciplinary escalation involving pediatrics, radiology, pediatric cardiology, otorhinolaryngology, intensive care, and cardiothoracic surgery.

Comparison with published literature

The clinical course of this infant is consistent with published experience showing that double aortic arch can produce clinically significant tracheal obstruction and that symptomatic patients often undergo surgical repair in infancy. In a large surgical series of complete vascular rings, double aortic arch represented a major proportion of operated patients, and trends over time included increased use of cross-sectional imaging for anatomical definition before surgery [13]. A multicenter study of children undergoing vascular-ring division demonstrated generally favorable long-term outcomes, while emphasizing that respiratory symptoms may persist in some patients after repair [14]. A contemporary review of congenital vascular rings similarly emphasizes the potential for airway and esophageal compression and the importance of appropriate surgical management [15].

The severity of airway compromise in the present case is particularly notable because the minimum tracheal diameter was only 1.7 mm. Earlier physiologic assessment of symptomatic infants with vascular rings demonstrated substantial preoperative airway dysfunction and improvement after repair, although residual abnormalities could remain in the early postoperative period [10]. A published report of double aortic arch also illustrates that airway obstruction can be a major clinical manifestation [16]. Anaesthetic airway management in infants with double aortic arch may present additional challenges [12]. The immediate postoperative improvement in the present infant is therefore clinically coherent with the published experience and provides additional support for the vascular compression mechanism.

The differential diagnosis is also supported by the literature. Congenital tracheal stenosis is most often associated with complete tracheal rings [5,6], while pulmonary artery sling has a strong association with intrinsic tracheal stenosis and complete rings [7,8]. The present case instead demonstrated a complete double-aortic-arch ring without a reported anomalous left pulmonary artery. Thus, the case illustrates how careful evaluation of vascular anatomy can prevent critical extrinsic compression from being mislabeled as primary congenital tracheal stenosis.

Multidisciplinary management and postoperative considerations

Management of symptomatic vascular rings requires coordination between radiology, pediatric cardiology, pediatric cardiothoracic surgery, pediatrics, intensive care, and airway specialists. In this case, early ENT assessment excluded an upper-airway cause, cross-sectional imaging identified the vascular abnormality, CT angiography defined the surgical anatomy, and the infant was transferred to a tertiary pediatric cardiology service when the severity of airway obstruction increased.

The rapid clinical improvement after vascular-ring release was an important outcome. Stridor and work of breathing improved, oxygen support was discontinued, and the infant was discharged without stridor at rest and with adequate breastfeeding. Nevertheless, postoperative surveillance remains appropriate because persistent or dynamic airway abnormalities may occur after prolonged vascular compression [10,11,14]. The planned pulmonology and otorhinolaryngology follow-up was therefore appropriate despite the favorable immediate outcome.

Limitations

Several limitations should be acknowledged. No bronchoscopy report was available, so a subtle intrinsic tracheal abnormality cannot be excluded with absolute certainty. Similarly, postoperative cross-sectional airway imaging or objective postoperative airway measurements were unavailable; therefore, the postoperative improvement reported here is clinical, consisting of resolution of stridor and work of breathing, discontinuation of supplemental oxygen, and improved feeding, rather than documented normalization of the tracheal lumen. The available records also did not provide standardized preoperative respiratory parameters such as serial blood gases or quantitative respiratory scores, and the operative report did not specify the exact vascular structure divided. These limitations restrict detailed quantitative comparison of airway status before and after repair.

The laboratory findings were not suggestive of a significant systemic inflammatory response, with C-reactive protein remaining <1 mg/L on both sampled dates. The differential count on July 22 showed a low absolute neutrophil count and elevated eosinophil count according to the laboratory's stated ranges; these findings were noted in the report and were not considered explanatory for the mechanical airway obstruction. The principal diagnostic conclusion is based on the documented clinical course, CT and CT angiographic anatomy, and postoperative response rather than on laboratory testing.

## Conclusions

Double aortic arch should be considered in infants with persistent or unexplained stridor, particularly when upper-airway examination is unrevealing and symptoms worsen with crying or feeding. In this infant, critically reduced tracheal caliber was demonstrated at the level of a complete vascular ring formed by a double aortic arch, and the marked improvement following vascular-ring release supports extrinsic vascular compression as the principal mechanism of airway obstruction. Dedicated CT angiography was essential for identifying the double-arch anatomy, defining branch-vessel origins, demonstrating the relationship between the vascular ring and the airway, and guiding multidisciplinary surgical management. Careful distinction between extrinsic vascular compression, pulmonary artery sling, and intrinsic congenital tracheal stenosis is important because these entities have different anatomical substrates and treatment strategies.
